# LBMNet: a hybrid multi-scale CNN–Mamba framework for enhanced 3D stroke lesion segmentation in MRI

**DOI:** 10.3389/fmed.2026.1759114

**Published:** 2026-02-09

**Authors:** Zhejun Kuang, Xingxue Yan, Jiaxuan Yu, Dawen Sun, Jian Zhao, Lei Sun

**Affiliations:** 1 College of Computer Science and Technology, Changchun University, Changchun, China; 2 Jilin Provincial Key Laboratory of Human Health Status Identification Function & Enhancement, Changchun, China; 3 Key Laboratory of Intelligent Rehabilitation and Barrier-Free for the Disabled, Changchun University, Ministry of Education, Changchun, China; 4 College of Artificial Intelligence, Nankai University, Tianjin, China

**Keywords:** brain stroke segmentation, deep learning, hybrid architecture, Mamba, medical imaging, MRI, multi-scale convolution, state space model

## Abstract

**Introduction:**

Brain stroke is one of the leading causes of death and disability worldwide, and accurate lesion segmentation from MRI is critical for clinical diagnosis and treatment planning. However, existing methods struggle with the high variability of stroke lesions in size and morphology. In particular, they fail to detect small lesions due to the limited receptive fields of CNNs and the computational inefficiency of Transformer-based approaches. To address these challenges, we propose LBMNet, a novel CNN–Mamba network that integrates multi-scale convolutional encoding with Mamba-based decoding.

**Methods:**

Owing to the high heterogeneity of stroke lesions, the encoder design employs a top-down LSC module to capture cross-scale representations. The decoder designs the BSC-Mamba (Bidirectional Spatial Context Mamba) model, integrating bidirectional state space modeling with adaptive spatial convolutions to enhance local feature information while modeling global dependencies with linear complexity. Furthermore, asymmetric adaptive gated feature fusion (BAGF) bridges the semantic gap by selectively merging encoder and decoder features, suppressing redundant information whilst highlighting critical lesion details.

**Results:**

Extensive experiments on two benchmark datasets demonstrate state-of-the-art performance, achieving Dice coefficients of 67.57% on ATLAS v2.0 and 82.03% on ISLES 2022. Compared with existing CNN, Transformer, and hybrid models, LBMNet shows significant improvements in small lesion segmentation. This study presents a robust and efficient framework with strong clinical potential for accurate stroke lesion segmentation across diverse lesion sizes and morphologies.

## 1 Introduction

Stroke is a leading global cause of disability and mortality (1, 2). According to the World Stroke Organization, it has become the third leading cause of death globally, with stroke-related fatalities projected to rise by nearly 50% between 2020 and 2050. Etiologically, stroke is categorized into ischemic and hemorrhagic types, with ischemic

stroke being predominant, accounting for approximately 80% of all cases. In clinical practice, rapid and precise lesion segmentation is crucial for treatment planning, formulating rehabilitation strategies, and assessing prognosis (3). However, stroke lesions on medical images often exhibit pronounced heterogeneity, posing formidable challenges for manual segmentation. Manual annotation depends heavily on the operator's expertise, introduces subjectivity, and is both time-consuming and inconsistent (4). Consequently, developing efficient and accurate automated segmentation techniques has become an urgent priority in this domain.

**Figure 1:**
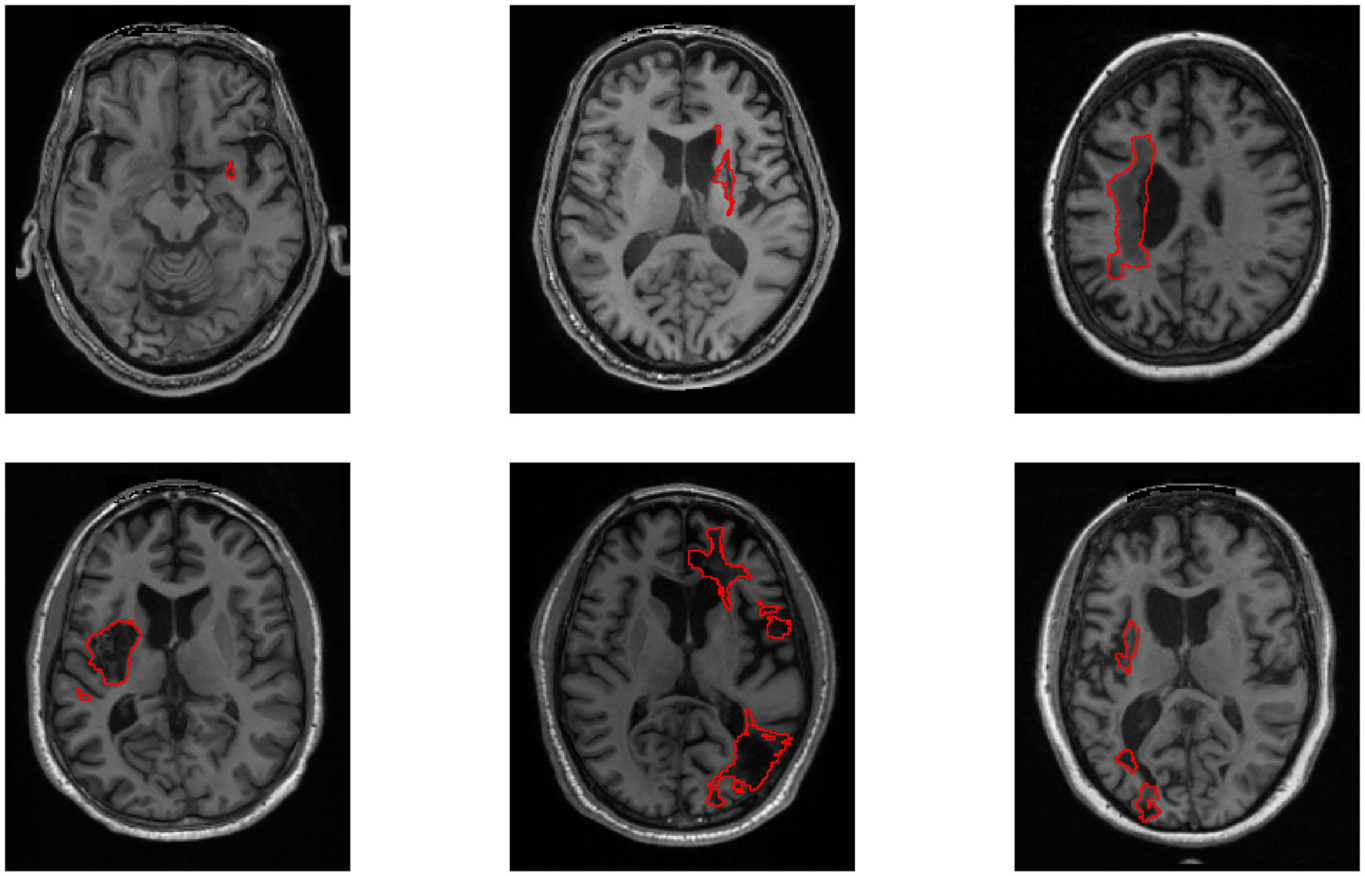
Representative examples of stroke lesions with diverse sizes, shapes, and anatomical locations. Red regions indicate lesions (ATLAS v2.0).

Despite the rapid progress of deep learning in medical image segmentation (5), automatic segmentation of stroke lesions remains challenging. Stroke lesions vary extensively in morphology: they can manifest as small ischemic regions with a volume under 10 cm^3^—highly susceptible to omission—or as large, irregular hemorrhagic lesions with indistinct boundaries that are difficult to delineate. As illustrated in Figure 1, stroke lesions exhibit substantial variability in size, morphology, and anatomical location, ranging from small focal lesions to large irregular regions, which poses significant challenges for accurate segmentation.

Due to the limited receptive fields, the CNN methods cannot capture global context information well. For example, U-Net (6) and its variants can obtain high Dice for medium-to-large lesions, but often miss small lesions due to their limitation of fine-grained features capture. On the other hand, the transformer-based architectures, motivated by the self-attention, can capture global context information well. However, these methods require large-scale annotated datasets, which are hard to obtain. While labeled samples are abundant in stroke imaging, lesion boundaries are often ambiguous, which would lead transformers to overfit and consequently perform poorly in boundary delineation (7). Recently, CNN-Transformer hybrids such as TransUNet (8), Swin-UNETR (9) alleviate the above issues to a certain degree; however, they adopt naive skip connections through simply concatenating features, which would bring redundant information, feature conflicts, and degrade robustness under multi-scale lesion conditions.

These limitations indicate that current methods are unable to effectively capture global context information and simultaneously localize the boundaries of small and irregular stroke lesions accurately. CNNs are good at local feature extraction, while Transformers are good at global modeling. Still, these two approaches, used individually or even in a trivial combination, are unable to adaptively integrate the heterogeneous characteristics of stroke lesions.

To address these challenges, we propose LBMNet—a three-dimensional stroke lesion segmentation hybrid network tailored for highly heterogeneous lesion-scale scenarios. During the encoding phase, LBMNet employs an LSC module that captures global contextual information through dual large-kernel convolutions, while progressively refining local details via small-kernel cascading processing. This effectively characterizes lesion features ranging from minute to extensively distributed. During decoding, the BSC-Mamba module builds upon the Mamba state space modeling framework (10) to construct local spatial augmentations. Combined with bidirectional state space scanning, this approach efficiently models long-range dependencies while preserving spatial continuity. These multi-level features are further coordinated through the BAGF (Bidirectional Adaptive Gate Fusion) module in the skip connection. By dynamically integrating complementary information from the encoder and decoder via attention mechanisms, the model achieves precise segmentation of minute and morphologically complex stroke lesions.

To address these challenges, we propose a multi-scale CNN–Mamba fusion network (LBMNet) tailored for stroke lesion segmentation. Unlike Transformers with quadratic computational complexity (O(n^2^)), our model leverages Mamba's state-space mechanism (10) to achieve this goal efficiently. The main contributions are as follows:

1. To address the extreme scale variation of stroke lesions-ranging from minute localized foci to extensive diffuse areas-we designed a “coarse-to-fine” LSC module. Unlike standard convolutional neural networks constrained by fixed receptive fields, this architecture employs dual-branch large-kernel convolutions to concurrently capture global semantic information, followed by cascaded small-kernel convolutions to refine local structural details. This dynamic adaptation mechanism ensures precise feature capture across the entire lesion size spectrum, effectively mitigating omissions caused by lesion heterogeneity.
2. To address the inevitable information loss during global context modeling and sequence flattening, we propose BSC-Mamba. As standard state-space models necessitate flattening 3D volumes into 1D sequences, this process disrupts the voxel-level neighborhood structure essential for defining irregular boundaries. BSC-Mamba's design effectively compensates for this information loss by integrating Adaptive Spatial Convolution (ASC) to pre-enhance local structural representations. Through bidirectional state space processing, it simultaneously captures global long-range dependencies with linear complexity while preserving voxel integrity for precise segmentation.
3. We have implemented Adaptive Gated Feature Fusion (BAGF). To overcome semantic gaps and feature conflicts arising from highly heterogeneous lesions, conventional methods employ direct concatenation for jump connections, which frequently introduces noise due to mismatched encoder and decoder representations. BAGF employs an asymmetric attention strategy: spatial attention in the encoder and channel attention in the decoder enable autonomous feature selection and dynamic fusion. This design ensures only lesion-relevant information is transmitted, effectively suppressing redundant and superfluous feature information.

Extensive experiments conducted on two benchmark datasets, ATLAS v2.0 and ISLES 2022, demonstrate that LBMNet substantially outperforms existing CNN-based methods, Transformer-based methods, and hybrid architectures across multiple evaluation metrics. LBMNet achieves a Dice score of 67.57% on ATLAS v2.0 and 82.03% on ISLES 2022. Compared with state-of-the-art baselines, the proposed model exhibits superior segmentation performance, particularly in detecting small lesions.

## 2 Related work

### 2.1 CNN-based methods

#### 2.1.1 U-Net and its variants in general medical segmentation

Convolutional neural networks are considered the most appropriate models for medical image segmentation. U-Net successfully segments medical images using an encoder-decoder design and skip-connection architecture. R2U-Net (11) enhances feature representation by utilizing recurrent convolution and residual modules; Attention U-Net (12). Unlapping irrelevant features of skip connections in an attention gating mode. The 3D U-Net proposed by Çiçek et al. directly works for volumetric data by fully leveraging the spatial continuity of 3D scans. However, convolutional neural networks cannot capture the long-term dependency involved in segmenting morphologically complex lesions. Due to the large size variations of the lesions, small lesions do not segment well, and large lesions only partially perform segmentation, which is considered a major problem in segmentation failure.

#### 2.1.2 Multi-scale convolutional strategies

To adapt to the scale variations of visual targets, inception networks (13), Res2Net (14), and HRNet (15) employ parallel or multi-branch convolutions for feature extraction from 2D classification and detection tasks. While these methods demonstrate excellent performance, they exhibit limitations in stroke lesion segmentation, failing to capture fine-grained lesion features due to a lack of voxel consistency. In our LSC module complete preservation of semantic information through a dual mechanism of global modeling (large-kernel convolutions) and local refinement (small-kernel convolutions).

#### 2.1.3 CNN approach for stroke detection

There are also some works reported in studies suggesting CNN architectures for stroke lesion segmentation. For instance, Dolz et al. (16) presented a dense multi-path CNN that passes features of different scales in parallel. It is able to capture rich 2D features but cannot make good use of the highly 3D spatial context. Another method, ML-Net, is strong in terms of distance-based metrics (HD95) but not stable in the Dice score, especially for small lesions. Unlike FRPNet (17), which employs a feature refinement pyramid to enhance the segmentation accuracy, these methods are based on fixed-scale kernels and thus are limited to dealing with the high heterogeneity of stroke lesion sizes. These methods still lack the adaptability to cover the wide range of lesion sizes. It is still a challenging task to accurately segment both small lesions and large diffuse lesions (18).

### 2.2 Transformer-based methods

Transformers, based on self-attention, give powerful global context modeling for medical segmentation. TransUNet (8) combined Transformers with CNNs to achieve a better result in segmentation boundaries. Swin-UNETR (9) also improved feature encoding by using a hierarchical Swin Transformer, which gave excellent results for multi-organ segmentation, which requires very large labeled datasets. In stroke imaging, where annotated samples are scarce and lesion boundaries are often ambiguous, Transformers may underfit and fail to segment boundaries (7) accurately. Their quadratic complexity (O(n^2^)) also limits scalability in the high-resolution 3D data. In the stroke segmentation problems, they lead to fine boundary segmentation and instability in small-lesion segmentation issues. While hybrid CNN-Transformer models partly solve the problems, their simple concatenation of skip connections can result in feature redundancy and cross-scale instability.

### 2.3 State space models and the Mamba architecture

As the sequence modeling ability is being adopted, the State Space Model (SSM) has received a lot of attention recently. Mamba (10) models global dependency linearly (O(n)); therefore, this eliminates the quadratic bottleneck of Transformers. Recently, researchers have applied Mamba to medical image segmentation. Earlier works VMamba (19) and VM-U-Net (20) successfully adapted the Mamba block for vision tasks. Recently UMamba (21), SegMamba (22) and HCMUNet (23) were proposed for medical image analysis. The common approach in these approaches is to serialize the 2D/3D patches of the image to 1D sequences as the input of Mamba blocks. This “flattening” process may also destroy the 3D spatial connection of voxels and lose fine-grained information used to draw boundaries.

In this work, we propose our method, the BSC-Mamba decoder, which introduces a local convolutional enhancement prior to the state space modeling and preserves local features as much as possible, accompanied by a bidirectional scanning strategy. While previous applications of Mamba focus on improving efficiency, our method aims at balancing global context and local spatial details to provide more robust performance for various lesion shapes.

## 3 Methods

**Figure 2:**
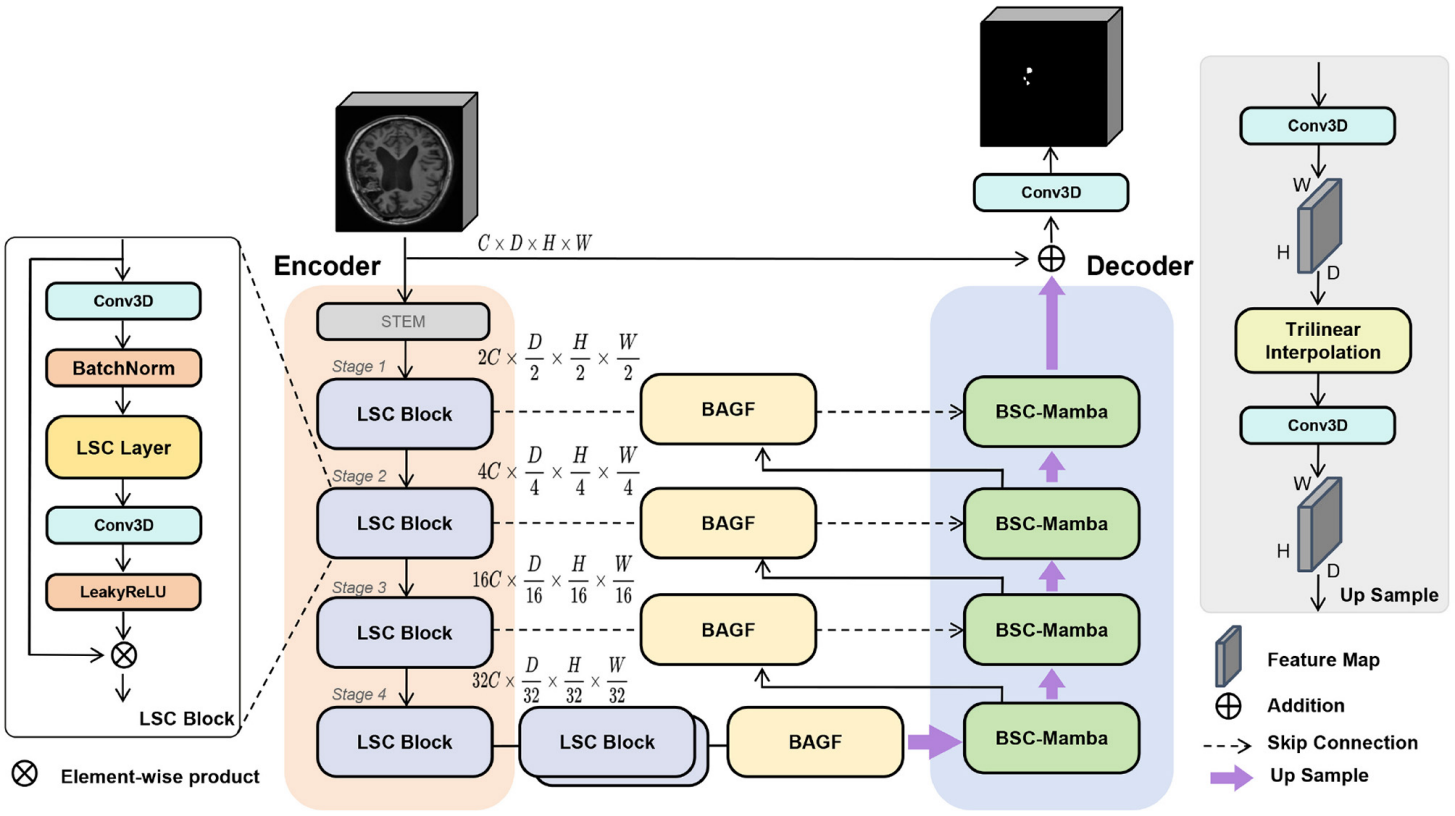
LBMNet consists of a multi-scale convolutional encoder (LSC), a locally enhanced bidirectional Mamba decoder (BSC-Mamba), and an adaptive feature fusion module (BAGF).

We present LBMNet and its architecture shown in Figure 2. The network architecture includes three main components: Encoder, Adaptive Feature Fusion (BAGF) module, and Decoder. For feature extraction in the encoding process, multiple LSC Block layers are stacked in the encoder. The medical image is (C, D, H, W), where C, D, H, and W are the number of channels, depth, height, and width, respectively. The input image goes through a STEM convolution layer for initial feature extraction, which keeps spatial structure while enhances channel expressiveness for multi-level feature extraction. The image will be fed into an encoder consisting of 4 stages, and these stages are alternatively stacked with LSC Blocks. When the network goes deeper, the features are gradually squeezed: spatially downsampling while channels become deeper. Since the information will be lost when downsampling the feature maps, BAGF modules on skip connections will connect the features of the encoder generated layer by layer with the features of the decoder output and transfer them to the corresponding lower decoder stage. The decoder adopts a progressive upsampling-based architecture, and BSC-Mamba modules are used to combine local and global information to recover spatial resolution. The final segmentation output will be obtained by a 3D convolution layer that maps the features of fused layers.

### 3.1 Encoder based on multi-scale dynamic receptive fields

**Figure 3:**
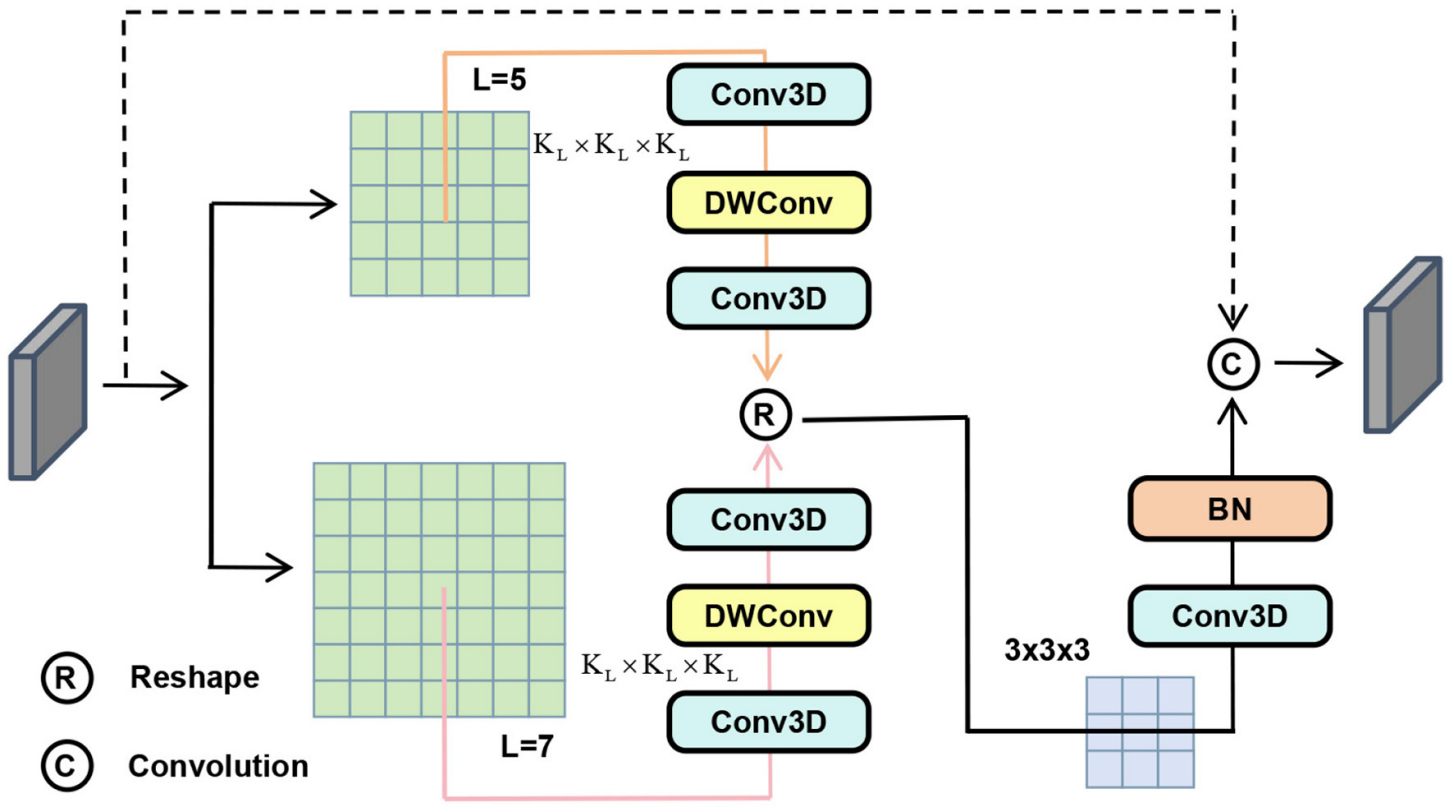
LSC adopts dual large kernels with 3 × 3 refinement for hierarchical “large-to-small” feature extraction.

Stroke lesions exhibit a high degree of heterogeneity at different scales. Fine-grained features need to be extracted for accurate boundary segmentation in small lesions (< 10 cm^3^), while the global context needs to be captured using larger receptive fields in relatively larger lesions (>50 cm^3^). However, standard convolutional neural networks employing fixed-size kernels suffer from inherent limitations: they either fail to detect minute lesions due to excessive stride sizes or are unable to fully capture comprehensive information from large lesions. To address this scale-sensitivity issue, to capture such scale-dependent heterogeneity, as shown in Figure 3, we design the “Large-to-Small Convolution” module in the encoder in a similar way to a large kernel in RepLKNet (24) and ConvNeXt (25). The most distinctive contribution of the LSC module is that it explicitly introduces a “coarse-to-fine” hierarchical refinement process, while previous models solely depend on a single large-kernel branch.

LSC initially extracts extensive multi-scale contextual information using parallel large-kernel branches, followed by immediate feature refinement through a dedicated small-kernel path. Specifically, the input feature map *x* is first processed through two parallel large-kernel depthwise separable convolutions (with kernel sizes 5^3^ and 7^3^):


$$\begin{array}{l} F^{\left(l\right)}=\underset{s\in \left\{5,7\right\}}{\sum }\text{DWConv}3{\text{D}}_{s}({\text{Conv}}_{1\times 1\times 1}\left(x\right)) \end{array}$$ (1)

Subsequently, small-kernel convolutions are introduced for local refinement:


$$\begin{array}{l} y=\text{Con}{\text{v}}_{3\times 3\times 3}\left(F^{\left(l\right)}\right) \end{array}$$ (2)

Finally, feature stability is ensured through residual paths and batch normalization:


$$\begin{array}{l} Y=x+\text{BN}({\text{Conv}}_{1\times 1\times 1}\left(y\right)) \end{array}$$ (3)

The LSC module combines dual large cores with refined small cores, specifically addressing lesion-scale heterogeneity. It provides a powerful mechanism for extracting robust, multi-scale features tailored to the specific distribution characteristics of stroke lesions.

### 3.2 Global–local state space module

**Figure 4:**
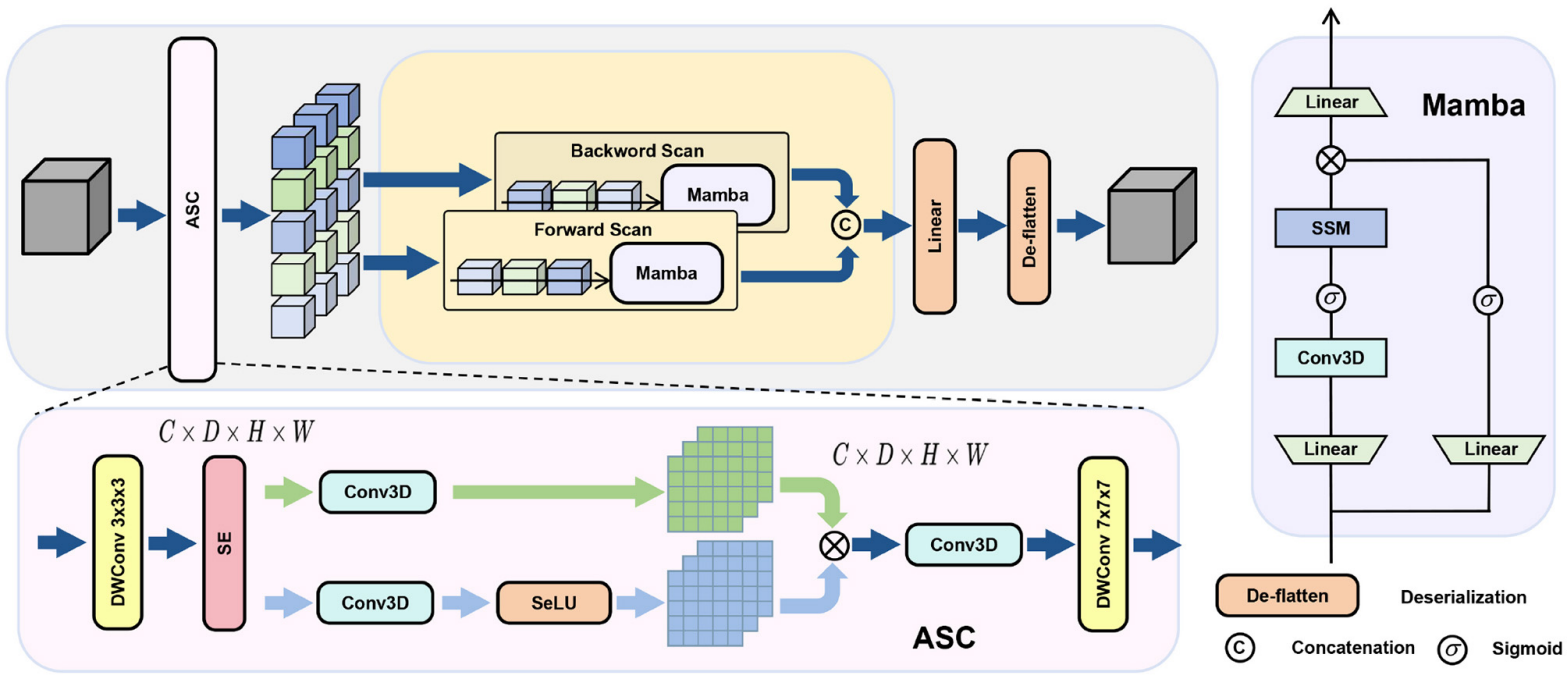
BSC-Mamba module combines bidirectional state space modeling with local space context enhancement.

Segmentation of stroke lesions is typically applied on large-scale, irregularly shaped 3D data, thus requiring models with a global receptive field to model long-range dependencies. Transformer models are feasible to model long-range dependencies, while the self-attention cost is (O(n^2^)). For typical 3D medical images, this leads to prohibitive computational cost and memory consumption. Therefore, we use the Mamba architecture to model global context with linear complexity (O(n)) and greatly reduce the computational overhead of global context modeling. Thus, it is extremely scalable in modern medical imaging. On the other hand, training Mamba directly on images is not desirable. Mamba takes data as one-dimensional sequences, which implies that 3D feature maps need to be “unrolled.” However, this “unrolling” process inevitably degrades spatial information at the voxel level, preventing the standard Mamba architecture from effectively delineating the irregular boundaries characteristic of stroke lesions. To address this, we propose the Bidirectional Spatial Context Mamba (BSC-Mamba) decoder, which incorporates Adaptive Spatial Convolution (ASC) prior to bidirectional global scanning. This enhances local features while efficiently modeling global information. The BSC-Mamba module comprises two main stages, as shown in Figure 4.

#### 3.2.1 Adaptive spatial convolution (ASC) for local enhancement

Before sequence flattening, the input feature map *x* is processed by the ASC module. This module is designed to enhance local spatial information that may be lost during the serialization process. Initially, a depth-separable 3 × 3 × 3 convolution is applied to capture direct neighborhood relationships. Then, the SE attention mechanism adapts the channel feature information based on the local spatial context, effectively “preprocessing” the features before passing them to the global Mamba stage. This approach ensures the encoding and preservation of fine details, such as the edges of minute lesions.


$$\begin{array}{l} x_{se}=f_{\text{SE-Attn}}\left({\text{Conv}}_{3\times 3\times 3}\left(x\right)\right)⊗{\text{DWConv}}_{3\times 3\times 3}\left(x\right) \end{array}$$ (4)

Subsequently, a gated convolutional unit and a large kernel (7 × 7 × 7) depthwise convolution further expand the local receptive field, thereby creating a robust feature representation with local perceptual capabilities *y*.


$$\begin{array}{l} y=x+{\text{Conv}}_{7\times 7\times 7}\left({\text{Conv}}_{1\times 1\times 1}\left(x_{se}\right)⊗\phi \left({\text{Conv}}_{1\times 1\times 1}\left(x_{se}\right)\right)\right) \end{array}$$ (5)

#### 3.2.2 Bidirectional state space modeling for global context

After local enhancement by ASC, the 3D feature map *y* is flattened into a one-dimensional sequence *y*_*seq*_. Standard unidirectional Mamba scans are limited in that they can only capture context from preceding elements. This approach is inadequate for spatial data, where the spatial context is isotropic. The features of a lesion at a given voxel depend on its global context, which must be considered to capture the full spatial relationship. Therefore, we adopted a bidirectional scanning mechanism to process the sequence in both forward and backward directions:


$$\begin{array}{l} y_{\text{fwd}}=\text{Mamba}\left(y_{seq}\right) \end{array}$$ (6)

$$\begin{array}{l} y_{\text{bwd}}=\text{reverse}\left(\text{Mamba}\left(\text{reverse}\left(y_{seq}\right)\right)\right) \end{array}$$ (7)

Forward scanning captures dependencies from the “beginning” to the “end” of the flattened sequence, while backward scanning tracks dependencies from the “end” to the “beginning.” By concatenating *y*_fwd_ and *y*_bwd_, we ensure that the feature representation at each position is informed by the complete global context from both directions along the scan axis. This bidirectional approach is essential for accurately modeling complex structures, such as stroke lesions.

Finally, the concatenated features are projected back to the original channel dimension, reshaped into 3D volumetric data, and subsequently linked through residual connections to preserve the integrity of the information.


$$\begin{array}{l} \text{output}=\text{reshape}\left(W_{f}\left[y_{\text{fwd}};y_{\text{bwd}}\right]\right)+x \end{array}$$ (8)

### 3.3 Bidirectional feature fusion

As demonstrated by the U-Net, these skip connections help reintroduce high-resolution information into the decoding path. However, a significant “semantic gap” persists between encoder and decoder features. While encoder features from deeper layers provide highly semantic information, their weak spatial resolution makes it challenging to precisely localize minute lesions and delineate their boundaries accurately. Conversely, the decoder's highly spatially resolved information is semantically weaker and more susceptible to noise.

This leads to poor segmentation results and may even cause over-segmentation. If the two types of feature information are directly concatenated, conflicts and redundant information will arise, resulting in segmentation errors.

**Figure 5:**
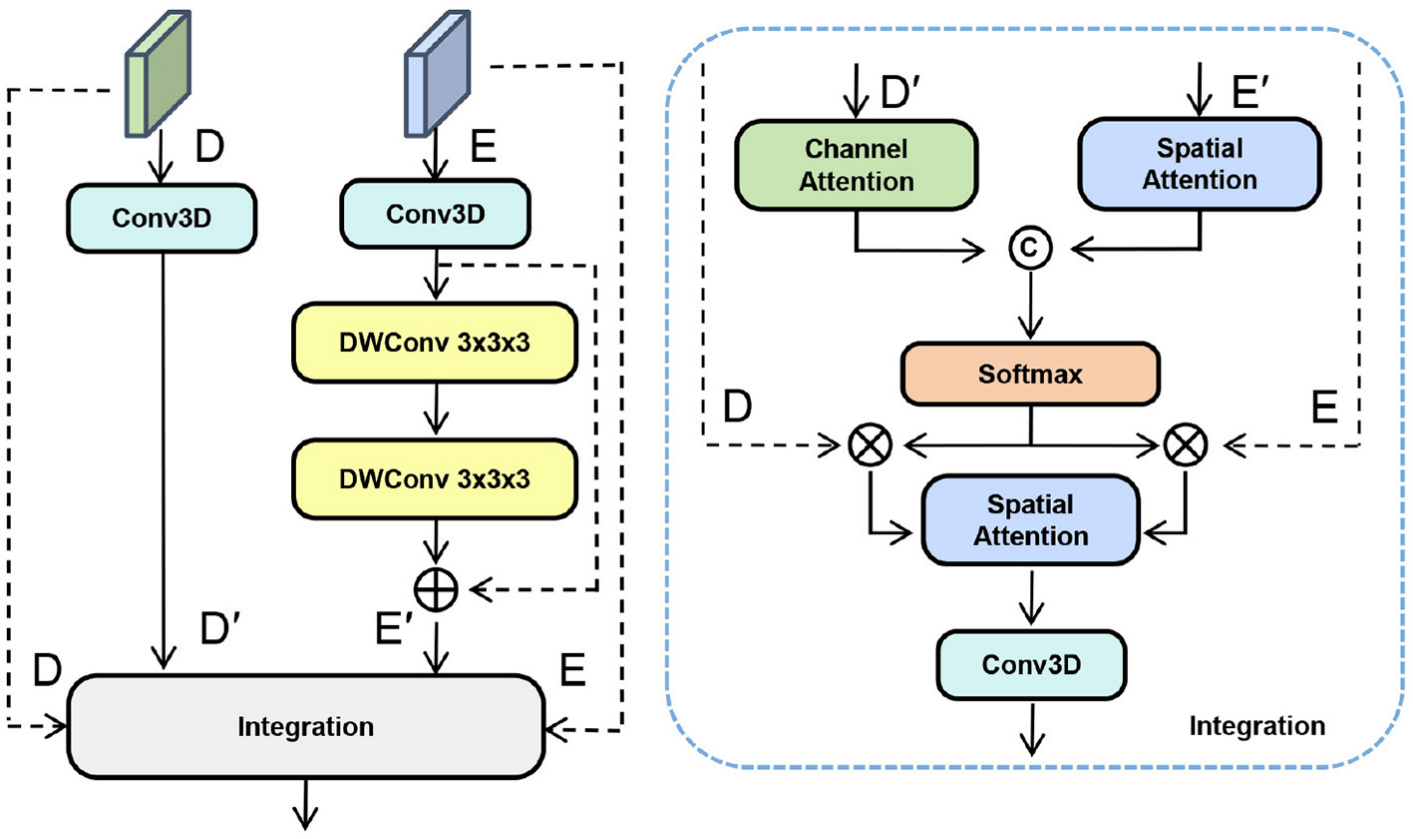
The BAGF module applies channel and spatial attention to encoder–decoder features and adaptively fuses them.

Therefore, we propose the adaptive fusion (BAGF) module shown in Figure 5, which efficiently utilizes effective information from both the encoder and decoder. Encoder features contain multi-scale contextual information but lack spatial information; decoder features contain spatial information but may contain noise. To address this, we apply a spatial attention mechanism in the encoder path to suppress irrelevant contextual regions while enhancing effective spatial information. Simultaneously, a channel attention mechanism is employed in the decoder path to highlight lesion-relevant feature channels while suppressing noisy ones. Finally, these two optimized feature streams are selectively fused to achieve the desired integration.

First, the input encoder features (*E*) and decoder features (*D*) are aligned along a common channel dimension and subsequently augmented. The encoder features undergo further refinement through a lightweight residual block, enhancing local feature representations. These refined features are denoted as *E*′ and *D*′, respectively. Next, an asymmetric attention mechanism is applied. The spatially attention-enhanced encoder features *F*_enc_ and the channel attention-enhanced decoder features *F*_dec_ are generated as follows:


$$\begin{array}{l} F_{\text{enc}}=SA\left(E^{\prime }\right),\;F_{\text{dec}}=CA\left(D^{\prime }\right) \end{array}$$ (9)

Among these, *SA* represents spatial attention, and *CA* is channel attention. Then, these two enhanced feature maps are fused through a gating mechanism. This mechanism adaptively calculates weight coefficients α and β to balance the feature information from both paths.


$$\begin{array}{l} \left[\alpha ,\beta \right]=\text{Softmax}\left(\text{Gate}\left(\left[F_{\text{enc}},F_{\text{dec}}\right]\right)\right) \end{array}$$ (10)

$$\begin{array}{l} F_{\text{fused}}=\alpha \cdot F_{\text{enc}}+\beta \cdot F_{\text{dec}} \end{array}$$ (11)

Finally, the fused feature map passes through the final convolutional layer to generate the output *O*, which is then fed into the next decoder stage.


$$\begin{array}{l} O={\text{Conv}}_{1\times 1\times 1}\left(F_{\text{fused}}\right) \end{array}$$ (12)

The BAGF module significantly enhances the model's ability to segment complex lesions by effectively integrating multi-level features. This module improves segmentation accuracy by effectively emphasizing lesion regions while suppressing irrelevant information.

### 3.4 Loss function

In medical image segmentation, the significant foreground-background class imbalance is addressed by a hybrid loss function that combines Dice loss and weighted cross-entropy (CE) loss. This hybrid approach is widely used in current segmentation tasks.


$$\begin{array}{l} L\left(y,ŷ\right)=\alpha \left[-E_{i\in \text{Ω}}\overset{C}{\underset{c=1}{\sum }}y_{i,c}\log\left({ŷ}_{i,c}\right)\right]\\ \;+\beta \left(1-\frac{2\underset{i\in \text{Ω}}{\sum }y_{i}{ŷ}_{i}+\epsilon }{\sum_{i\in \text{Ω}}y_{i}+\sum_{i\in \text{Ω}}{ŷ}_{i}+\epsilon }\right) \end{array}$$ (13)

Here, ŷ represents the predicted probability distribution, and *y* denotes the true label. α and β are the weighting coefficients for cross-entropy and Dice loss, respectively. ϵ is the smoothing factor for numerical stability, Ω is the pixel set, and *C* is the total number of categories.

### 3.5 Evaluation metrics

To quantitatively evaluate the segmentation performance of LBMNet and compare it with other methods, we employed several standard metrics widely used in medical image segmentation, including the Dice coefficient, Intersection over Union (IoU), Recall, F2 score, and the 95th percentile Hausdorff Distance (HD95). All metrics were computed based on true positives (TP), false positives (FP), and false negatives (FN). The Dice coefficient measures the overlap between the predicted and ground truth regions, the IoU (or Jaccard index) evaluates the ratio of intersection to union, Recall quantifies the proportion of correctly identified positive voxels, and the F2 score emphasizes Recall, while HD95 measures the 95th percentile bidirectional Hausdorff distance between the predicted contour (*P*) and the ground truth (*G*). Their definitions are as follows:


$$\begin{array}{l} \text{Dice}=\frac{2\times \text{TP}}{2\times \text{TP}+\text{FP}+\text{FN}} \end{array}$$ (14)

$$\begin{array}{l} \text{IoU}=\frac{\text{TP}}{\text{TP}+\text{FP}+\text{FN}} \end{array}$$ (15)

$$\begin{array}{l} \text{Recall}=\frac{\text{TP}}{\text{TP}+\text{FN}} \end{array}$$ (16)

$$\begin{array}{l} \text{F2}=\left(1+2^{2}\right)\times \frac{\text{Precision}\times \text{Recall}}{\left(2^{2}\times \text{Precision}\right)+\text{Recall}} \end{array}$$ (17)

$$\begin{array}{l} \text{HD95}\left(P,G\right)=\max{\left(K_{95th}(\underset{g\in \partial G}{\min}||p-g{||}_{2}\right)}_{p\in \partial P},\\ \;K_{95th}{\left(\underset{p\in \partial P}{\min}||g-p{||}_{2}\right)}_{g\in \partial G}) \end{array}$$ (18)

## 4 Results and discussion

### 4.1 Datasets

This is shown through experiments on two publicly available stroke lesion segmentation datasets: ATLAS v2.0 and ISLES 2022. ATLAS v2.0 is a dataset of single-modality T1-weighted (T1W) MR images from chronic-phase stroke patients, extending the previous public dataset of the same name (26). The dataset consists of 1,271 cases, comprising 655 publicly available training cases, 300 public test cases with annotations covered up (hidden annotations), and 316 new test cases that are unpublished. The training dataset released by the organizers was split into training, validation, and test sets with 8:1:1 ratio.

ISLES 2022 is a dataset of ischemic stroke lesions in acute and sub-acute stages using multimodal MRI imaging, including FLAIR, DWI, and ADC images. Although the DWI and ADC images were registered to each other, the FLAIR images were not. Since the DWI and ADC images are most effective at representing the ischemic stroke lesions, we used these two images and discarded the FLAIR image. We used 250 cases from ISLES 2022 dataset (27). To guarantee a rigorous and fair comparison with state-of-the-art baselines, and to strictly align with the widely accepted evaluation protocol of the nnUNet (28) framework, we maintained a consistent data partitioning strategy. Consequently, the dataset was randomly split into training, validation, and test sets with a fixed ratio of 8:1:1.

### 4.2 Implementation details

All the experiments in this study are conducted with PyTorch in NVIDIA GeForce RTX 3090. We employ Python 3.10 and CUDA 11.8 to integrate LBMNet into nnUNet (28). We use PyTorch's Auto Mixed Precision (AMP) to perform mixed precision training (FP16). AMP can decrease the GPU memory consumption while keeping good numerical stability.

We adopt the standardized preprocessing pipeline of the nnUNet framework, which has been validated in numerous medical imaging challenges. The preprocessing pipeline includes the following steps:

- Resampling: all volumetric data were resampled to the median voxel spacing of 1.0 × 1.0 × 1.0 mm^3^. The images were processed using cubic spline interpolation, while segmentation masks utilized nearest-neighbor interpolation to maintain the integrity of the labels.
- Intensity normalization: for each MRI modality, we apply *z*-score normalization based on foreground voxels (non-zero regions).
- Trim: trim the volume to a non-zero bounding box while preserving a 5-voxel margin.

Reasons for adopting nnUNet preprocessing: this protocol ensures alignment with cutting-edge medical image segmentation techniques, enabling equitable comparisons with baseline models.

During training, the initial learning rate was set to 0.001, the weight decay coefficient to 3 × 10^−5^, and the optimizer selected was SGD with a momentum parameter of 0.99. Deep supervision was enabled. The model was trained for 300 epochs with a batch size of 2. The loss function employed a hybrid Dice-cross-entropy loss. Mamba utilized a state dimension (*d*_*s*_*tate*) of 16 and an expansion factor of 2. To ensure fair comparison, all baseline methods employed the same configuration.

### 4.3 Analysis of experimental results

#### 4.3.1 Quantitative and qualitative analysis

In order to assess LBMNet's performance, we trained the model on the ATLAS v2.0 dataset and compared LBMNet's results with a conventional CNN-based network [U-Net (6), 3DUnet (29), U-Net++ (30)], a Transformer-based network UNETR, TransUNet, SwinUNet, and recently proposed hybrid models [STU-Net (31), FRPNet, UMamba]. As shown in Table 1, LBMNet receives a Dice score of 67.57% as compared to the baseline U-Net (Dice = 48.34%), which improves by 17.51%. In comparison with the previous state-of-the-art UMamba (Dice = 63.31%), LBMNet improves by 4.26%.

**Table 1:** Comparison of different models on the ATLAS v2.0 dataset.

**Methods**	**Dice (%)**	**IoU (%)**	**F2 (%)**	**Recall (%)**	**HD95 (px)**
3Dunet	50.06	41.39	51.12	57.07	39.57
U-Net++	45.85	34.07	50.86	42.62	40.88
SwinUNet	51.10	40.00	57.23	62.47	34.52
TransUNet	50.01	46.14	55.47	58.29	42.86
UNETR	56.23	44.11	57.43	58.33	45.44
STU-Net	59.92	44.65	62.39	64.00	38.30
FRPNet	60.16	47.06	64.35	67.74	36.20
Umamba	63.31	49.64	68.31	68.25	29.06
LBMNet	67.57	52.03	72.39	69.38	22.32

This impressive performance boost is due to our proposed architectural design. First, the performance gap with respect to CNN models shows the effectiveness of our LSC encoder. By dynamically modeling features from various receptive fields, the LSC module effectively alleviates the limitation of great variation of lesion-size existing in fixed-kernels CNN models. Second, compared with the Transformer-based models (note they are strong at modeling global context but are likely to ignore fine-grained information), LBMNet shows the effectiveness of our method. Specifically, the cooperation between BSC-Mamba decoder and BAGF skip-connection module is strong. BSC-Mamba decoder is able to model effective global dependencies and local spatial contexts efficiently, while BAGF module can filter and aggregate most informative features from both encoder and decoder adaptively. Thus, the information loss in pure Transformer model is alleviated. The global-connected segmentation result of BSC-Mamba decoder can help to refine the local segmentation results to accurately segment the lesion boundary.

**Figure 6:**
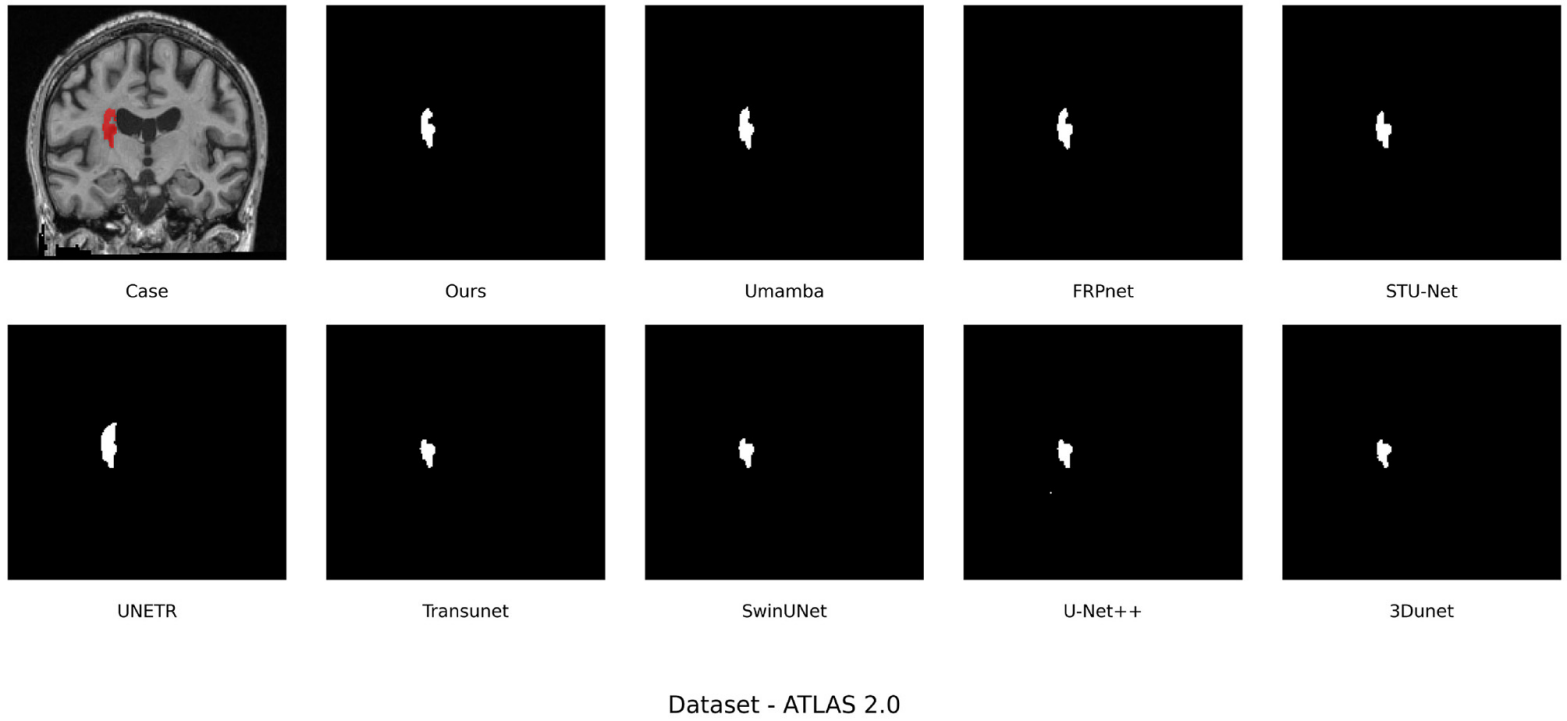
ATLAS v2.0 dataset visualization qualitative analysis.

In addition, as can be seen from the four metrics, LBMNet shows the best performance. As shown in Figure 6, the IoU of 52.03% is the largest, which means the segmentation result is closer to the ground truth. What is more interesting is that the F2-score of LBMNet achieves 72.39% and the Recall rate of LBMNet is 69.38%. In a medical application, high recall indicates that the model is unlikely to lead to false negative, which means the model is unlikely to miss the actual lesion area and further misdiagnose the disease. Because the lesion area to segment is very complex, having a globally connected segmentation result helps to minimize the false negatives in local segmentation.

In addition, in Figure 6, we give the qualitative results and the results are visually convincing for the quantitative results above. Specifically, it is obvious that compared with pure CNN-based model, U-Net++, due to the limitation of receptive field, the global continuity of large lesions cannot be well captured, which leads to the undersegmentation. While, TransUNet, although it is better at modeling global information of lesions, tends to smooth the global connection or even omit the detailed boundary information, which leads to unsatisfactory segmentation. Globally connected segmentation result of U-Net++ and local detailed segmentation result of TransUNet are used to refine the final segmentation result of LBMNet.

#### 4.3.2 Diagnostic analysis of different and sizes of lesions

To evaluate the model's adaptability to lesion size, we specifically categorized the ATLAS v2.0 test set into three groups based on lesion volume: small lesions (< 10 cm^3^), medium lesions (10–50 cm^3^), and large lesions (>50 cm^3^). Due to the limited sample size of the ISLES 2022 dataset, we did not conduct lesion size stratification experiments on it. The detailed results for ATLAS v2.0 are provided in Table 2.

**Table 2:** Performance evaluation of the model on the ATLAS v2.0 test set for different lesion sizes.

**Size (cm^3^)**	**Proportion (%)**	**Dice (%)**	**IoU (%)**	**Recall (%)**
< 10	61.8	58.47	48.31	66.52
10–50	20.6	70.06	53.94	69.74
>50	17.6	74.18	59.51	74.02

In the segmentation of small lesions, we obtain satisfactory model performance, and the Dice coefficient and recall are 58.47 and 66.52%, respectively. The above results demonstrate that LBMNet can still discover and retain most of the lesion areas for minute targets. The hierarchical strategy of LSC module “coarse-grained capture and then fine-grained refinement” is very important to avoid losing the local details, which are usually ignored by ordinary convolutional layers. As expected, the evaluation metrics increase gradually with the increase of lesion volume. For the medium-sized lesions, the Dice score achieves 70.06%. And for the large lesions, the Dice score can also reach an excellent 74.18%. The above consistent and robust performance for all the lesion sizes further demonstrates the strength of our model on handling such large scale variation for stroke lesions.

**Figure 7:**
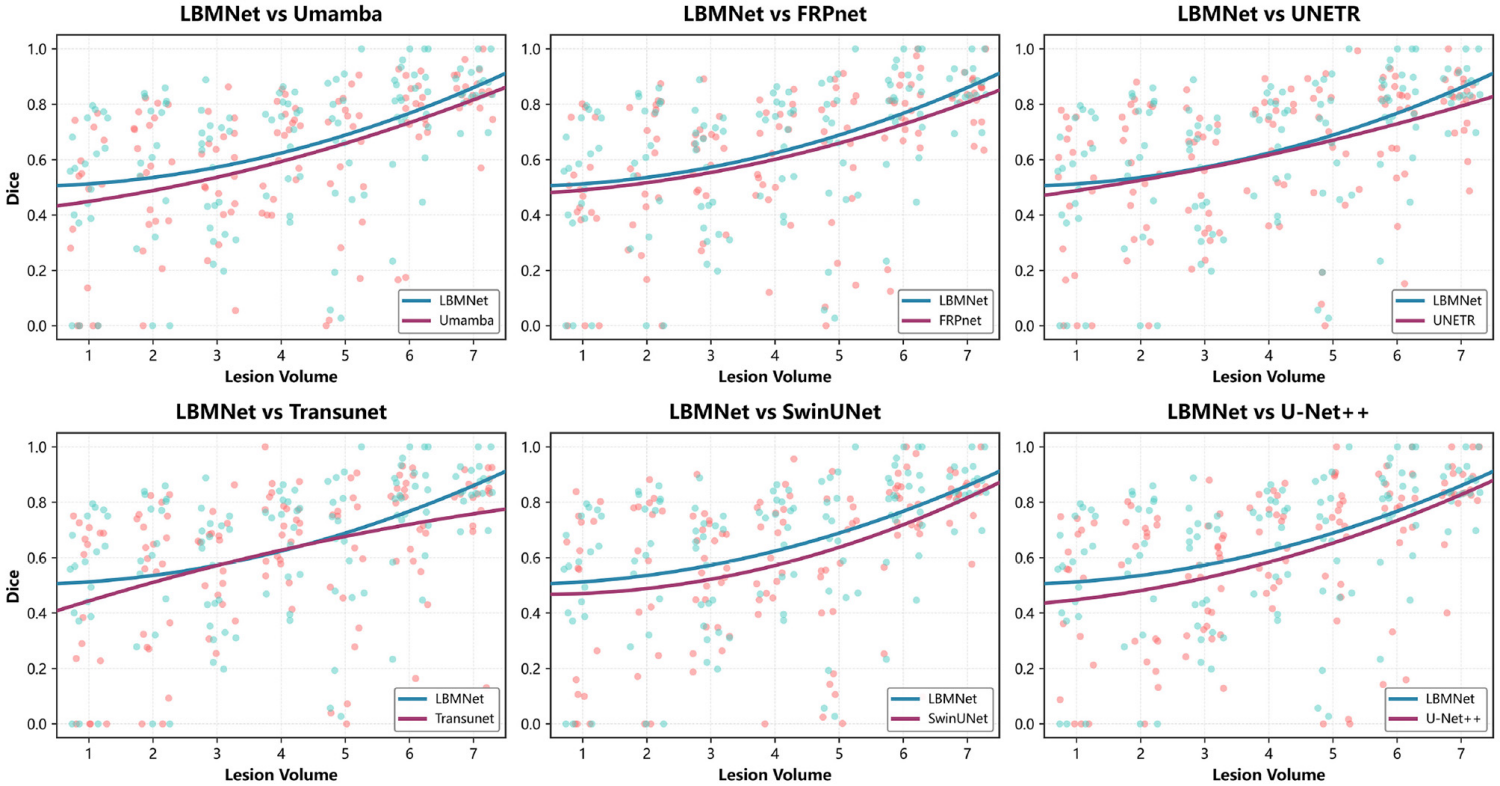
The figure compares Dice scores of LBMNet and other models across lesion volumes.

The effectiveness of LBMNet is also verified in the comparison shown in Figure 7. Specifically, for the case of small lesions (< 10 cm^3^). In addition to Umamba, our LBMNet also achieves state-of-the-art performance. It is of great importance that missing small lesions are highly risky, and the effectiveness of our model in maintaining details is remarkable due to its multi-scale feature extraction. For medium-sized lesions, our model still has an advantage over the models, while some models also get similar performance for this range. For large lesions, our model has its own advantage, which shows the ability of our model to integrate global context with local details and keep the segmentation part intact. To rigorously validate the model's robustness on small lesions (< 10 cm^3^), we conducted a comparative analysis against state-of-the-art methods (Table 3). Consequently, we utilized Recall (Sensitivity) and Hausdorff Distance (HD95) as more clinically relevant metrics to experimentally validate the performance on the small lesion subset. As shown in Table 3, while baseline models like SwinUNet struggle with a low Recall of 47.55%, LBMNet achieves a superior Recall of 66.52%. This indicates a significantly lower rate of missed diagnoses, which is critical for early stroke screening. Furthermore, our HD95 is reduced to 24.12 px (compared to < 29 px for UMamba), demonstrating that LBMNet preserves the topological structure of small lesions more accurately than competitors, even when the Dice metric fluctuates.

**Table 3:** Performance comparison on the small lesion subset (< 10 cm^3^) of ATLAS v2.0.

**Methods**	**Dice (%)**	**IoU (%)**	**F2 (%)**	**Recall (%)**	**HD95 (px)**
3D U-Net	39.15	27.65	43.10	45.20	42.18
U-Net++	41.50	30.15	45.80	48.25	39.50
SwinUNet	40.82	28.56	44.20	47.55	40.15
TransUNet	42.15	29.85	46.50	49.12	38.92
UNETR	38.45	26.90	42.10	45.30	43.20
STU-Net	47.92	35.88	52.85	54.20	33.56
FRPNet	49.65	37.12	54.10	56.33	31.20
Umamba	51.28	39.85	56.42	58.14	29.45
LBMNet (Ours)	58.47	48.31	63.85	66.52	24.12

**Figure 8:**
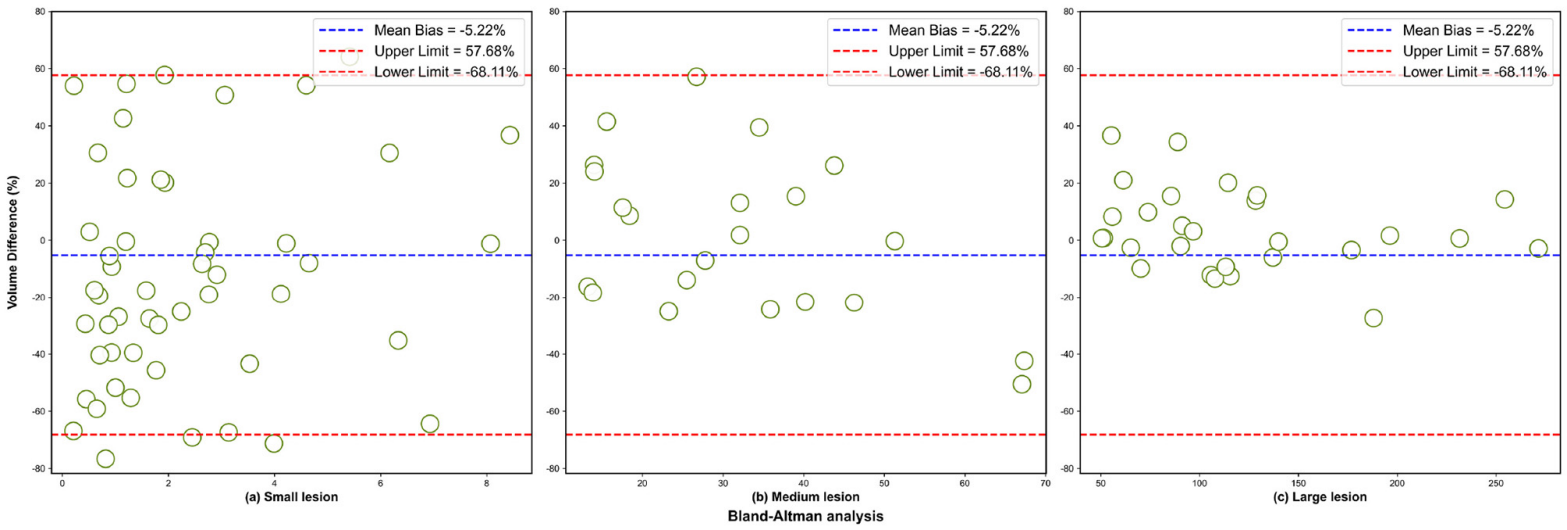
Bland–Altman plots for different lesion volumes: **(a)** small lesions, **(b)** medium lesions, and **(c)** large lesions.

To further assess the accuracy of volume segmentation, we conducted a Bland–Altman analysis to evaluate segmentation errors across lesions of various sizes, as illustrated in Figure 8. The Bland–Altman analysis revealed a mean bias of –5.22%, indicating a slight systematic underestimation of lesion volume. The 95% confidence interval ranged from –68.11 to 57.68%, with larger relative deviations mainly observed in small lesions. This bias was most pronounced in small lesions (Figure 8a), where boundary ambiguity likely contributed to larger relative volume discrepancies. However, even within this challenging subgroup, the majority of data points remained within the confidence interval. As the lesion size increased to medium (Figure 8b) and large (Figure 8c), the variability in segmentation error notably decreased, with data points clustering tightly around the mean bias line. This progressively improved consistency underscores the model's reliability for clinically relevant lesions. Overall, the Bland–Altman analysis confirms that LBMNet provides robust and accurate volume assessments across the full range of lesion sizes.

### 4.4 Experimental analysis on the ISLES 2022 dataset

In order to thoroughly evaluate the generalization ability and robustness of LBMNet, we extend the analysis to the ISLES 2022 dataset. Unlike ATLAS v2.0, the ISLES 2022 dataset provides MRI images containing stroke lesions in both acute and sub-acute phase. The stroke lesions are show greater signal heterogeneity and have less distinct boundaries, which challenges the model.

As shown in Table 4, LBMNet achieves a Dice coefficient of 82.03% and F2 score of 85.50%. Not only does its performance outperform other prominent CNNs (U-Net++ 68.10%), or even Transformer-based networks (UNETR 76.92%), but it also exhibits remarkable consistency across datasets with different characteristics. In addition, the HD95 decreases dramatically to 21.15 pixels, outperforming most competing models. Such an impressive improvement on boundary delineation accuracy further verifies the effectiveness of our proposed BAGF module, which can retain some spatial details via selecting salient features from both encoder and decoder adaptively and accurately segment the lesions with blurry boundaries.

**Table 4:** Comparison of different models on the ISLES 2022 dataset.

**Methods**	**Dice (%)**	**IoU (%)**	**F2 (%)**	**Recall (%)**	**HD95 (px)**
U-Net	56.34	45.47	59.47	60.80	36.35
3Dunet	58.06	48.39	59.12	67.07	39.57
U-Net++	62.85	50.07	67.86	59.62	35.88
SwinUNet	68.10	57.00	74.23	79.47	35.52
TransUNet	67.01	63.14	72.47	75.29	36.86
UNETR	73.23	61.11	74.43	75.33	33.44
STU-Net	76.92	61.65	79.39	81.00	27.31
FRPNet	77.16	64.03	79.33	79.74	29.20
Umamba	74.31	57.64	80.31	78.25	26.06
LBMNet	82.03	61.50	85.50	84.30	21.15

**Figure 9:**
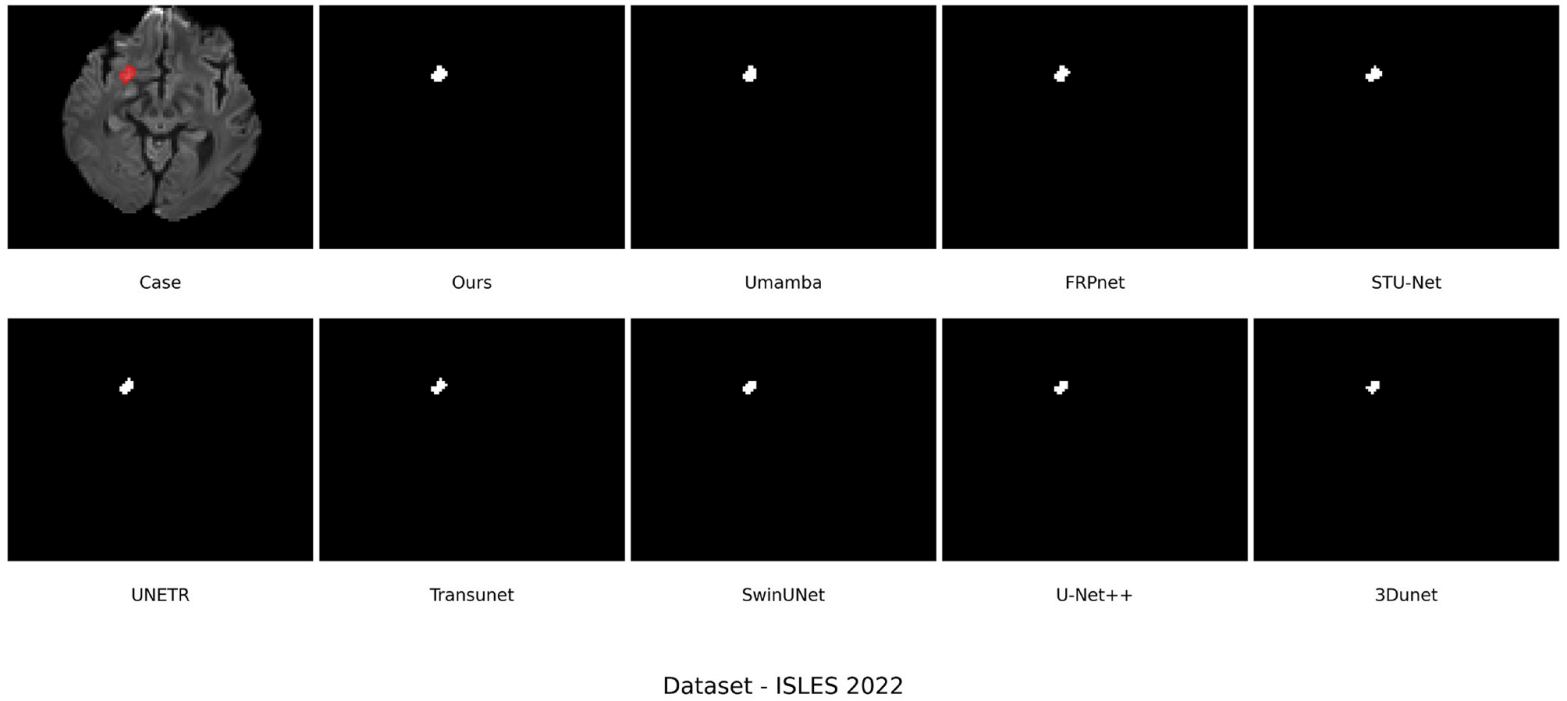
ISLES 2022 dataset visualization qualitative analysis.

As shown in Figure 9, qualitative analysis also reveals the benefits of LBMNet in the case of segmentation of small lesions. It can be seen from the figures that our model delineates the small lesion from the data. Severe under-segmentation by UMamba and SwinUNet, as well as under-segmentation by U-Net++, is evident in these visualizations. The strong global context provided by the BSC-Mamba decoder (bidirectional state space modeling) allows the model to segment small but highly intricate lesions. Furthermore, the convolution strategy of the LSC encoder makes the model sensitive to small targets. In conclusion, extensive evaluations on the ISLES 2022 dataset confirm that LBMNet is not solely fine-tuned for a particular data distribution, but instead exhibits strong generalization ability and robustness.

### 4.5 Robustness analysis via repeated random splits

While the fixed 8:1:1 split ensures fair comparison with benchmarks, the relatively small size of the test set (especially for ISLES 2022) may raise concerns regarding statistical reliability. To further validate the stability and generalization capability of LBMNet, we conducted an additional robustness analysis using 5 independent runs of repeated random splits. In this experiment, which is independent of the main benchmark comparison, the dataset was randomly shuffled and re-split into 8:1:1 for each run with different random seeds.

As presented in Table 5, the average performance across these five random splits remains highly consistent with our reported main results. Specifically, for the ISLES 2022 dataset, LBMNet achieved an average Dice score of 81.75% ± 1.45%, which is very close to the 82.03% reported in the fixed split. The low standard deviation (±1.45%) indicates that the model's performance is stable and not dependent on a specific data partition. Similarly, for ATLAS v2.0, the mean Dice score of 67.42% ± 1.24% confirms the robustness of our method against data variations.

**Table 5:** Robustness analysis results under five repeated random splits.

**Dataset**	**Dice (%)**	**IoU (%)**	**Recall (%)**	**HD95 (px)**
ATLAS v2.0	67.42 ± 1.24	51.88 ± 1.15	69.15 ± 2.10	22.95 ± 3.40
ISLES 2022	81.75 ± 1.45	61.30 ± 1.60	84.10 ± 1.90	21.90 ± 3.80

### 4.6 Ablation study

#### 4.6.1 Components ablation

**Figure 10:**
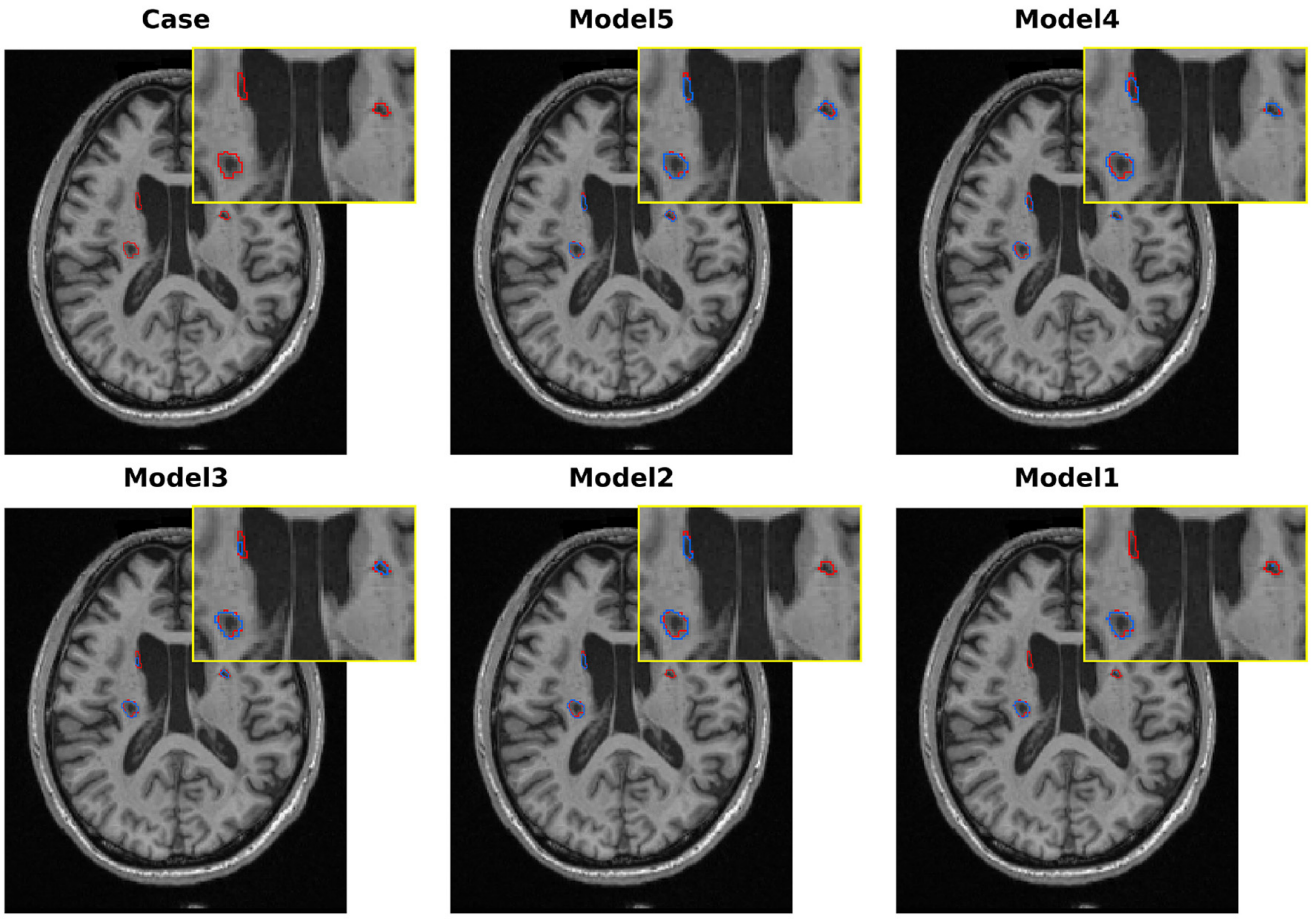
Qualitative comparison of various models in melting research on the ATLAS v2.0 dataset.

To further analyze the effectiveness of each proposed component, we conducted a series of ablation experiments on the ATLAS v2.0 dataset. These experiments were performed in independent runs and were primarily used to analyze the relative performance contributions of each component. We use 3D U-Net as a baseline model and successively add our approach, namely multi-scale LSC encoder, BSC-Mamba decoder, and adaptive BAGF skip-connection module, to evaluate the improvement in both quantitative and qualitative results shown in Table 6 and Figure 10, respectively.

**Table 6:** Performance comparison of different components.

**Method**	**LSC encoder**	**Mamba decode**	**BAGF**	**Dice (%)**	**IoU (%)**
Model 1	×	×	×	50.06	41.39
Model 2	✓	×	×	60.09	47.01
Model 3	×	✓	×	63.11	51.38
Model 4	✓	✓	×	65.03	53.23
Model 5	✓	✓	✓	67.57	54.97

Our baseline model, the standard 3D U-Net, obtains Dice = 50.06% and IoU = 41.39%. As shown in Figure 10, the model can roughly locate the lesions, but it clearly undersegments them and fails to capture the complicated boundaries of the lesions. These results demonstrate the limitations of traditional convolutional networks in modeling the variety of sizes and irregular shapes of stroke lesions.

To alleviate the limitations of the baseline in multi-scale feature extraction, we replaced the conventional encoder with our proposed LSC encoder (Model 2). The single modification leads to a remarkable improvement in the Dice score (+10.03 percentage points, 60.09%). As shown in Figure 10, the qualitative results also support this improvement, where the model outlines a more complete region of the primary lesion area. These results demonstrate the effectiveness of the LSC module in learning features from various receptive fields, which are crucial for modeling both core and peripheral areas of lesions. However, the model is still unable to detect the lesions in the opposite hemisphere.

To validate the effectiveness of global context modeling, we swapped out the baseline decoder for BSC-Mamba (Model 3). With this setup, we obtained Dice score of 63.11%. From Figure 10, we can see that Model 3 was able to model bilateral lesions, which demonstrated the power of BSC-Mamba to model long-range dependencies and utilize distant spatial information. Meanwhile, the model lost its fine-grained local information and thus led to slight under-segmentation in boundary regions.

After independently validating the effectiveness of the encoder and decoder, we concatenate the encoder and decoder to build Model 4. This new model takes advantage of LSC's ability to extract multi-scale local features and BSC-Mamba's ability to integrate global–local information. The Dice coefficient achieves 65.03%. Visualization results show that the lesion region is more completely captured by Model 4, and the contour is sharper and clearer. However, directly fusing features may cause over-segmentation at blurred boundaries, which means that conflicting or redundant features are probably forwarded via skip connections.

To address the suboptimal feature fusion issue, we further developed the BAGF module to obtain the complete LBMNet (Model 5). Specifically, BAGF module adaptively chooses and fuses salient features from encoder and decoder pathways while suppressing noise and redundant information. With the further improvement of feature fusion, the final model achieves the best segmentation performance as shown in (Model 5: Dice = 67.57%, IoU = 54.97%). As shown in Figure 10, the segmentation masks generated by Model 5 are most accurate. That is, the boundaries of segmentation masks are clear and there is neither over-segmentation nor under-segmentation. The above results also demonstrate that the BAGF module plays a key role in integrating multi-scale and global representations to obtain robust and accurate segmentation results.

#### 4.6.2 LSC module ablation

To analyze the influence of our particular design choices in the LSC module, we perform an ablation study in Table 7. The baseline model in Table 7 is different from Model 1 in Figure 9. Specifically, the baseline is the complete LBMNet model (Model 5), where we replace its LSC module with a typical 3D residual block with only 3^3^ convolutions. With this controlled setup, the only part of the network that varies from the baseline is the LSC module, meaning that if the LSC design choices lead to any improvement, this excludes the possibility that other components of the decoder or skip connections might be causing it.

**Table 7:** Experimental results of LSC ablation.

**Method**	**Configuration**	**Kernel**	**Dice (%)**	**IoU (%)**
Baseline	Res-block	Single 3^3^	58.21	44.39
Model A	Kernel only	Single 5^3^	62.15	50.90
Model B	Kernel only	Single 7^3^	64.93	52.78
Model C	Kernel only	Single 9^3^	64.52	52.41
Model D	Dual-branch refinement	No 3^3^	66.17	54.39
Ours	LSC module	5^3^+7^3^+3^3^	67.57	54.97

Replacing the baseline 3^3^ convolutional kernel with a single large kernel (Models A and B) resulted in substantial performance improvements, with gains of +3.94% for 5^3^ and +6.72% for 7^3^. These findings confirm the importance of large receptive fields in effectively capturing stroke lesions. However, increasing the kernel size from 7^3^ to 9^3^ (Model C) provided only a marginal gain of +0.19% in Dice score, accompanied by higher computational costs. More importantly, a single large kernel struggles to balance global context with local details. In contrast, the dual-branch design, which combines 5^3^ and 7^3^ convolutional kernels (Model D), significantly outperformed any single-kernel configuration, achieving a Dice score of 66.17%. This highlights the superiority of explicitly modeling features across multiple scales, rather than relying on a single large convolutional kernel. Our final LSC module design, which incorporates a 3^3^ refinement path after the dual-branch output (Ours), achieves the highest Dice score of 67.57%. The +1.4% improvement over Model D strongly supports our core “coarse-to-fine” hypothesis: capturing broad context with large kernels, followed by refinement with small kernels, is the optimal strategy.

#### 4.6.3 BSC-Mamba module ablation

To rigorously validate the design of our BSC-Mamba module, we conducted two sets of targeted ablation experiments.

First, our goal is to analyze the independent and synergistic advantages of two parts in BSC-Mamba: the ASC module in local enhancement and the bidirectional state-space model in global modeling. We designed four variants on top of a standard convolutional decoder baseline. The baseline (Baseline) uses LBMNet with a basic 3D convolutional decoder (Table 8) shows the necessity of these two parts. With only the ASC module (1.14 Dice gain), the network can still greatly improve local feature representations and thus proves the advantage of its design to preserve local information. With only Bi-directional Mamba (2.22 Dice gain), the network demonstrates the necessity of global context modeling. But the performance of Bi-directional Mamba is limited due to the information loss from sequence flattening. The whole BSC-Mamba that applies ASC in local preprocessing and Bi-SSM in global modeling obtains the best performance.

**Table 8:** Ablation study results of the BSC-Mamba module on stroke segmentation tasks.

**Method**	**SSM**	**ASC**	**Dice (%)**	**IoU (%)**
Baseline	×	×	64.01	52.76
Only-ASC	×	✓	65.15	53.28
Only-SSM	✓	×	66.13	53.86
Ours	✓	✓	67.57	54.97

Next, we evaluated the necessity of the proposed bidirectional scanning strategy by comparing it with alternative scanning methods, as illustrated in the Table 9. Transitioning from unidirectional to bidirectional scanning (our approach) produced a substantial performance improvement, with the Dice score increasing by 1.9%. This result highlights the critical role of reverse contextual information in accurately segmenting non-causal objects. Further extending the approach to quad-directional scanning yielded negligible additional gains in Dice score while introducing higher computational overhead. These findings strongly support the effectiveness of the bidirectional scanning strategy, which captures nearly all essential global information without the unnecessary computational burden of multi-axis scanning.

**Table 9:** Ablation study results of the BSC-Mamba module on stroke segmentation tasks.

**Strategy**	**Description**	**Dice (%)**	**IoU (%)**
One-way Mamba	Forward (D)	65.67	53.76
Two-way Mamba	Bidirectional (D)	67.57	54.97
Four-way Mamba	Bidirectional (D + H)	65.31	53.36

#### 4.6.4 BAGF module ablation

**Figure 11:**
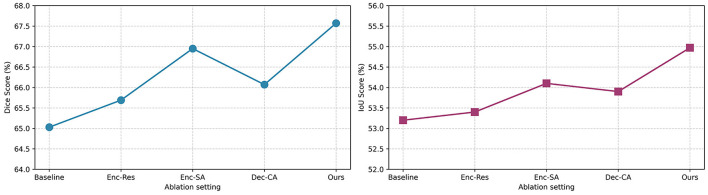
Experimental results of BAGF module ablation.

Test the performance of our proposed BAGF module and its components, we conducted an ablation study as follows: we started with a key baseline model in which we replaced the BAGF module with a concatenation operation following the design of the standard U-Net. Then, we successively added each of our proposed components to this baseline: Enc-Res: residual depthwise separable convolutional blocks added to the encoder path to improve feature representation; Enc-SA: spatial attention operation added to the encoder path; and Dec-CA: channel attention operation added to the decoder path. As illustrated in Figure 11, introducing the Enc-Res component alone improves the Dice score by 1.18%, demonstrating that strengthening local feature representations in the encoder path prior to feature fusion is advantageous. Incorporating the Enc-SA component results in an even greater gain, enhancing the Dice score by 2.02%, which confirms the effectiveness of selectively emphasizing spatially relevant regions within the encoder's coarse feature maps. Applying the Dec-CA component independently also produces a notable improvement, raising the IoU by 1.22%. This outcome highlights its capacity to refine decoder features by emphasizing lesion-relevant channels while suppressing noise-induced oversegmentation. The complete BAGF model achieves the best overall performance, with Dice score of 67.57% and an IoU of 54.97%. These results exceed the additive contributions of individual components, suggesting strong synergistic interactions among them. Collectively, these findings demonstrate that optimizing feature information within the skip-connection framework is essential for achieving accurate stroke lesion segmentation.

### 4.7 Discussion

We show that multi-scale convolutional encoding combined with Mamba-based decoding supported by adaptive feature fusion can substantially improve stroke lesion segmentation performance for small lesions (< 10 cm^3^) which have been highlighted as particularly challenging for recent ATLAS v2.0 benchmarks and systematic reviews (32–34). These lesions account for 61.8% of the ATLAS v2.0 test set and are often missed by other methods (35). Our model achieves a Dice score of 58.47%. This performance is attributed to the proposed architecture addressing some of the limitations in scale awareness and local detail preservation in current stroke models (36, 37) and improving semantic consistency under sequence-based decoders. We take LSC to the top-down hierarchical convolution by exploiting large convolution kernels to get global semantics, but then refining local structures with small kernels to avoid loss of fine-grained features of classical CNNs after downsampling. Dual-branch large-kernel performance gets 1.4%–5.4% performance gain with a single-kernel approach, and shows that multi-scale context is important for small lesions. Compared to Transformers' quadratic complexity and risk of overfitting on small-scale medical datasets, Mamba's linear complexity and selective state space mechanism are better suited for 3D medical data. However, directly applying it to 3D sequences disrupts local spatial relationships. Therefore, we incorporated an ASC module into BSC-Mamba to compensate for local structural information and adopted bidirectional scanning to integrate spatial dependencies from all directions. Experiments demonstrate that bidirectional scanning improves performance by 1.9% over unidirectional scanning, while further expanding scanning directions yields negligible gains, indicating that essential global relationships have been effectively captured. We achieve Dice scores of 67.57 and 82.03% on ATLAS v2.0 and ISLES 2022, respectively, validating this mechanism's effectiveness in medical imaging.

In order to reduce semantic differences between encoder and decoder features, we propose BAGF. It employs spatial attention to suppress background interference at the encoding stage and channel attention to highlight lesion-related features at the decoding stage, achieving adaptive fusion through a gating mechanism. This strategy achieves a 2.5% improvement over simple concatenation while significantly reducing boundary noise and segmentation errors—particularly crucial in stroke imaging where signal similarities often exist between different pathological tissues. Clinically, small lesions, though inconspicuous, hold significant prognostic value and are closely associated with cerebrovascular disease burden and future stroke risk (11, 38). The model's 66.52% recall rate for small lesions reduces the risk of missed diagnoses, facilitating early intervention. It demonstrates good generalization across different imaging modalities and disease stages. However, greater imaging variability exists in real-world clinical settings, necessitating prospective validation. Regarding the loss function, our experiments demonstrate that the standard hybrid loss (Dice + Weighted Cross-Entropy) is effective for this task. The Weighted Cross-Entropy term effectively addresses the class imbalance of small lesions, which is supported by the high Recall and low HD95 scores achieved on the small lesion subset (< 10 cm^3^). These results indicate that our current optimization strategy is sufficient. However, we acknowledge that boundary delineation remains a challenge, and future work may incorporate boundary-sensitive loss functions to further refine edge precision. While the data are manually annotated for fully supervised learning, scalability becomes limited, which semi-supervised methods may overcome (39, 40). The model's concept of “multiscale local modeling + efficient global modeling” also works in cross-task transferability, and the experiment indicates its potential for multi-organ and tumor segmentation (41).

## 5 Conclusion

In this work, we introduce LBMNet, a hybrid CNN–Mamba architecture developed to improve the segmentation of stroke lesions across a wide range of sizes and morphologies. By combining multi-scale convolutional encoding with efficient global sequence modeling and an adaptive fusion strategy, the model integrates fine structural details with broader contextual information while maintaining linear computational complexity. Specifically, LBMNet achieves a parameter count of 66.42 M and 54.80 G FLOPs, demonstrating a favorable balance between complexity and accuracy that fully meets the practical demands of automated lesion quantification and treatment planning—highlighting its strong deployment potential in clinical workstations. Future work will explore lightweight variants to further facilitate widespread adoption on resource-constrained devices.

Across two widely used benchmarks, ATLAS v2.0 and ISLES 2022, LBMNet consistently and significantly outperforms state-of-the-art CNN-based methods, Transformer-based methods, and hybrid architectures. The model achieves Dice scores of 67.57 and 82.03%, respectively, and shows marked improvements in detecting small lesions-a critical challenge in stroke neuroimaging. Ablation analyses further support the functional roles of the LSC encoder, the BSC-Mamba decoder, and the BAGF module, each contributing to more effective multi-scale representation, global–local feature integration, and encoder–decoder alignment. Although the results are promising, several challenges remain. Boundary delineation can still be difficult in cases where lesion margins are poorly defined (42, 43), and the reliance on fully annotated datasets limits broader clinical deployment. Moreover, while initial findings suggest that LBMNet may generalize beyond stroke imaging, further evaluation on additional neuroimaging tasks is needed.

Future development will focus on incorporating boundary-aware loss functions to enhance edge precision (44), exploring semi-supervised or weakly supervised learning to reduce annotation requirements, extending the framework to other brain segmentation tasks, and performing prospective clinical validation to assess performance under real-world imaging variability. Collectively, these results suggest that LBMNet provides an effective and efficient algorithm to achieve automated stroke lesion segmentation and demonstrates the merits of hybrid architectures that combine convolutional modeling with state-space mechanisms to advance neuroimaging analysis.

## Data Availability

The original contributions presented in the study are included in the article/supplementary material, further inquiries can be directed to the corresponding author.

## References

[1] Feigin VL Stark BA Johnson CO Roth GA Bisignano C Abady GG . Global, regional, and national burden of stroke and its risk factors, 1990–2019: a systematic analysis for the global burden of disease study 2019. Lancet Neurol. (2021) 20:795–820. doi: 10.1016/S1474-4422(21)00252-0 34487721 PMC8443449

[2] Katan M Luft A. Global burden of stroke. Semin Neurol. (2018) 38:208–11. doi: 10.1055/s-0038-1649503 29791947

[3] Maier O Menze BH Von Der Gablentz J Häni L Heinrich MP Liebrand M . ISLES 2015—a public evaluation benchmark for ischemic stroke lesion segmentation from multispectral MRI. Med Image Anal. (2017) 35:250–69. doi: 10.1016/j.media.2016.07.009 27475911 PMC5099118

[4] Ghafoorian M Karssemeijer N Heskes T Van Uden IWM Sanchez CI Litjens G . Location sensitive deep convolutional neural networks for segmentation of white matter hyperintensities. Sci Rep. (2017) 7:5110. doi: 10.1038/s41598-017-05300-5 28698556 PMC5505987

[5] Litjens G Kooi T Bejnordi BE Setio AAA Ciompi F Ghafoorian M . A survey on deep learning in medical image analysis. Med Image Anal. (2017) 42:60–88. doi: 10.1016/j.media.2017.07.005 28778026

[6] Ronneberger O Fischer P Brox T. U-Net: convolutional networks for biomedical image segmentation. In:Navab N Hornegger J Wells WM Frangi AF, editors. Medical Image Computing and Computer-Assisted Intervention-MICCAI 2015. vol. 9351. Cham: Springer International Publishing (2015). p. 234–41. doi: 10.1007/978-3-319-24574-4_28

[7] Shamshad F Khan S Zamir SW Khan MH Hayat M Khan FS . Transformers in medical imaging: a survey. Med Image Anal. (2023) 88:102802. doi: 10.1016/j.media.2023.102802 37315483

[8] Chen J Lu Y Yu Q Luo X Adeli E Wang Y . TransUNet: transformers make strong encoders for medical image segmentation. arXiv [Preprint]. (2021) arXiv:2102.04306. doi: 10.48550/arXiv.2102.04306

[9] Hatamizadeh A Nath V Tang Y Yang D Roth HR Xu D. Swin UNETR: swin transformers for semantic segmentation of brain tumors in MRI images. In:Crimi A Bakas S, editors. Brainlesion: Glioma, Multiple Sclerosis, Stroke and Traumatic Brain Injuries. vol. 12962. Cham: Springer International Publishing (2022). p. 272–84. doi: 10.1007/978-3-031-08999-2_22

[10] Gu A Dao T. Mamba: linear-time sequence modeling with selective state spaces. arXiv [Preprint]. (2023) arXiv:2312.00752. doi: 10.48550/arXiv.2312.00752

[11] Alom MZ Hasan M Yakopcic C Taha TM Asari VK. Recurrent residual convolutional neural network based on U-Net (R2U-Net) for medical image segmentation. arXiv [Preprint]. (2018) arXiv:1802.06955. doi: 10.48550/arXiv.1802.06955

[12] Oktay O Schlemper J Folgoc LL Lee M Heinrich M Misawa K . Attention U-Net: learning where to look for the pancreas. arXiv [Preprint]. (2018) arXiv:1804.03999. doi: 10.48550/arXiv.1804.03999

[13] Szegedy C Liu W Jia Y Sermanet P Reed S Anguelov D . Going deeper with convolutions. In: 2015 IEEE Conference on Computer Vision and Pattern Recognition (CVPR). Boston, MA: IEEE (2015). p. 1–9. doi: 10.1109/CVPR.2015.7298594

[14] Gao SH Cheng MM Zhao K Zhang XY Yang MH Torr P. Res2Net: a new multi-scale backbone architecture. IEEE Trans Pattern Anal Mach Intell. (2021) 43:652–62. doi: 10.1109/TPAMI.2019.2938758 31484108

[15] Wang J Sun K Cheng T Jiang B Deng C Zhao Y . Deep high-resolution representation learning for visual recognition. IEEE Trans Pattern Anal Mach Intell. (2021) 43:3349–64. doi: 10.1109/TPAMI.2020.2983686 32248092

[16] Dolz J Ben Ayed I Desrosiers C. Dense multi-path U-Net for ischemic stroke lesion segmentation in multiple image modalities. In:Crimi A Bakas S Kuijf H Keyvan F Reyes M Van Walsum T, editors. Brainlesion: Glioma, Multiple Sclerosis, Stroke and Traumatic Brain Injuries. vol. 11383. Cham: Springer International Publishing (2019). p. 271–82. doi: 10.1007/978-3-030-11723-8_27

[17] Wu Z Zhang X Li F Wang S Li J. A feature-enhanced network for stroke lesion segmentation from brain MRI images. Comput Biol Med. (2024) 174:108326. doi: 10.1016/j.compbiomed.2024.108326 38599066

[18] Liew SL Anglin JM Banks NW Sondag M Ito KL Kim H . A large, open source dataset of stroke anatomical brain images and manual lesion segmentations. Sci Data. (2018) 5:180011. doi: 10.1038/sdata.2018.11 29461514 PMC5819480

[19] Liu Y Tian Y Zhao Y Yu H Xie L Wang Y . VMamba: visual state space model. arXiv [Preprint]. (2024) arXiv:2401.10166. doi: 10.48550/arXiv.2401.10166

[20] Ruan J Li J Xiang S. VM-UNet: vision Mamba UNet for medical image segmentation. arXiv [Preprint]. (2024) arXiv:2402.02491. doi: 10.48550/arXiv.2402.02491

[21] Ma J Li F Wang B. U-Mamba: enhancing long-range dependency for biomedical image segmentation. arXiv [Preprint]. (2024) arXiv:2401.04722. doi: 10.48550/arXiv.2401.04722

[22] Xing Z Ye T Yang Y Liu G Zhu L. SegMamba: long-range sequential modeling Mamba for 3D medical image segmentation. In:Linguraru MG Dou Q Feragen A Giannarou S Glocker B Lekadir K et al., editors. Medical Image Computing and Computer Assisted Intervention-MICCAI 2024. vol. 15008. Cham: Springer Nature Switzerland (2024). p. 578–88. doi: 10.1007/978-3-031-72111-3_54

[23] Ma X Du Y Sui D. A U-shaped architecture based on hybrid CNN and Mamba for medical image segmentation. Appl Sci. (2025) 15:7821. doi: 10.3390/app15147821

[24] Ding X Zhang X Han J Ding G. Scaling up your kernels to 31 × 31: revisiting large kernel design in CNNs. In: 2022 IEEE/CVF Conference on Computer Vision and Pattern Recognition (CVPR). New Orleans, LA: IEEE (2022). p. 11953–65. doi: 10.1109/CVPR52688.2022.01166

[25] Liu Z Mao H Wu CY Feichtenhofer C Darrell T Xie S. A ConvNet for the 2020s. In: 2022 IEEE/CVF Conference on Computer Vision and Pattern Recognition (CVPR). New Orleans, LA: IEEE (2022). p. 11966–76. doi: 10.1109/CVPR52688.2022.01167

[26] Liew SL Lo BP Donnelly MR Zavaliangos-Petropulu A Jeong JN Barisano G . A large, curated, open-source stroke neuroimaging dataset to improve lesion segmentation algorithms. Sci Data. (2022) 9:320. doi: 10.1038/s41597-022-01401-7 35710678 PMC9203460

[27] Hernandez Petzsche MR De La Rosa E Hanning U Wiest R Valenzuela W Reyes M . ISLES 2022: a multi-center magnetic resonance imaging stroke lesion segmentation dataset. Sci Data. (2022) 9:762. doi: 10.1038/s41597-022-01875-5 36496501 PMC9741583

[28] Isensee F Jaeger PF Kohl SAA Petersen J Maier-Hein KH. nnU-Net: a self-configuring method for deep learning-based biomedical image segmentation. Nat Methods. (2021) 18:203–11. doi: 10.1038/s41592-020-01008-z 33288961

[29] Çiçcek Ö Abdulkadir A Lienkamp SS Brox T Ronneberger O. 3D U-Net: learning dense volumetric segmentation from sparse annotation. In:Ourselin S Joskowicz L Sabuncu MR Unal G Wells W, editors. Medical Image Computing and Computer-Assisted Intervention-MICCAI 2016. vol. 9901. Cham: Springer International Publishing (2016). p. 424–32. doi: 10.1007/978-3-319-46723-8_49

[30] Zhou Z Siddiquee MMR Tajbakhsh N Liang J. UNet++: redesigning skip connections to exploit multiscale features in image segmentation. IEEE Trans Med Imaging. (2020) 39:1856–67. doi: 10.1109/TMI.2019.2959609 31841402 PMC7357299

[31] Huang Z Wang H Deng Z Ye J Su Y Sun H . STU-Net: scalable and transferable medical image segmentation models empowered by large-scale supervised pre-training. arXiv [Preprint]. (2023) arXiv:2304.06716. doi: 10.48550/arXiv.2304.06716

[32] Deb P Bharadwaj Baru L Dadi K Raju SB. BeSt-LeS: benchmarking stroke lesion segmentation using deep supervision. In:Baid U Dorent R Malec S Pytlarz M Su R Wijethilake N et al., editors. Brainlesion: Glioma, Multiple Sclerosis, Stroke and Traumatic Brain Injuries. vol. 14668. Cham: Springer Nature Switzerland (2024). p. 23–35. doi: 10.1007/978-3-031-76160-7_3

[33] Ahmed R Al Shehhi A Hassan B Werghi N Seghier ML. An appraisal of the performance of AI tools for chronic stroke lesion segmentation. Comput Biol Med. (2023) 164:107302. doi: 10.1016/j.compbiomed.2023.107302 37572443

[34] Abbasi H Orouskhani M Asgari S Zadeh SS. Automatic brain ischemic stroke segmentation with deep learning: a review. Neurosci Inform. (2023) 3:100145. doi: 10.1016/j.neuri.2023.100145

[35] Luo J Dai P He Z Huang Z Liao S Liu K. Deep learning models for ischemic stroke lesion segmentation in medical images: a survey. Comput Biol Med. (2024) 175:108509. doi: 10.1016/j.compbiomed.2024.108509 38677171

[36] Tang F Ding J Quan Q Wang L Ning C Zhou SK. CMUNEXT: an efficient medical image segmentation network based on large kernel and skip fusion. In: 2024 IEEE International Symposium on Biomedical Imaging (ISBI). Athens: IEEE (2024). p. 1–5. doi: 10.1109/ISBI56570.2024.10635609

[37] Sun Y Dai D Zhang Q Wang Y Xu S Lian C. MSCA-Net: multi-scale contextual attention network for skin lesion segmentation. Pattern Recognit. (2023) 139:109524. doi: 10.1016/j.patcog.2023.109524

[38] Nguyen J Vo N Chang PD Chantaduly C Yu W Soun JE. Evaluation of small vessel disease burden on MRI and stroke outcomes. Front Neurol. (2025) 16:1628787. doi: 10.3389/fneur.2025.1628787 40703774 PMC12285601

[39] Wang Y Xiao B Bi X Li W Gao X. MCF: mutual correction framework for semi-supervised medical image segmentation. In: 2023 IEEE/CVF Conference on Computer Vision and Pattern Recognition (CVPR). Vancouver, BC: IEEE (2023). p. 15651–60. doi: 10.1109/CVPR52729.2023.01502

[40] Han K Sheng VS Song Y Liu Y Qiu C Ma S . Deep semi-supervised learning for medical image segmentation: a review. Expert Syst Appl. (2024) 245:123052. doi: 10.1016/j.eswa.2023.123052

[41] Li S Wang H Meng Y Zhang C Song Z. Multi-organ segmentation: a progressive exploration of learning paradigms under scarce annotation. Phys Med Biol. (2024) 69:11TR01. doi: 10.1088/1361-6560/ad33b5 38479023

[42] Lin Q Chen X Chen C Garibaldi JM. Boundary-wise loss for medical image segmentation based on fuzzy rough sets. Inf Sci. (2024) 661:120183. doi: 10.1016/j.ins.2024.120183

[43] Sun F Luo Z Li S. Boundary difference over union loss for medical image segmentation. In:Greenspan H Madabhushi A Mousavi P Salcudean S Duncan J Syeda-Mahmood T et al., editors. Medical Image Computing and Computer Assisted Intervention-MICCAI 2023. vol. 14223. Cham: Springer Nature Switzerland (2023). p. 292–301.

[44] Li S Zheng J Li D. Precise segmentation of non-enhanced computed tomography in patients with ischemic stroke based on multi-scale U-Net deep network model. Comput Methods Programs Biomed. (2021) 208:106278. doi: 10.1016/j.cmpb.2021.106278 34274610

